# Does hospital competition improve the quality of outpatient care? - empirical evidence from a quasi-experiment in a Chinese city

**DOI:** 10.1186/s13561-024-00516-4

**Published:** 2024-06-08

**Authors:** Zixuan Peng, Audrey Laporte, Xiaolin Wei, Xinping Sha, Peter C. Coyte

**Affiliations:** 1 School of Public Health, Southeast University, Suite 137, Kangjian Building, 87 Dingjiaqiao, Nanjing, Jiangsu 210009 China; 2 Institute of Health Policy, Management, and Evaluation, University of Toronto, Toronto, Ontario Canada; 3 Xiangya School of Medicine, Central South University, 172 Tongzipo Rd, Yuelu District, Changsha, Hunan 410013 China

**Keywords:** Hospital competition, Quality of care, China

## Abstract

**Background:**

Although countries worldwide have launched a series of pro-competition reforms, the literature on the impacts of hospital competition has produced a complex and contradictory picture. This study examined whether hospital competition contributed to an increase in the quality of outpatient care.

**Methods:**

The dataset comprises encounter data on 406,664 outpatients with influenza between 2015 and 2019 in China. Competition was measured using the Herfindahl-Hirschman index (HHI). Whether patients had 14-day follow-up encounter for influenza at any healthcare facility, outpatient facility, and hospital outpatient department were the three quality outcomes assessed. Binary regression models with crossed random intercepts were constructed to estimate the impacts of the HHI on the quality of outpatient care. The intensity of nighttime lights was employed as an instrumental variable to address the endogenous relationship between the HHI and the quality of outpatient care.

**Results:**

We demonstrated that an increase in the degree of hospital competition was associated with improved quality of outpatient care. For each 1% increase in the degree of hospital competition, an individual’s risk of having a 14-day follow-up encounter for influenza at any healthcare facility, outpatient facility, and hospital outpatient department fell by 34.9%, 18.3%, and 20.8%, respectively. The impacts of hospital competition on improving the quality of outpatient care were more substantial among females, individuals who used the Urban and Rural Residents Basic Medical Insurance to pay for their medical costs, individuals who visited accredited hospitals, and adults aged 25 to 64 years when compared with their counterparts.

**Conclusion:**

This study demonstrated that hospital competition contributed to better quality of outpatient care under a regime with a regulated ceiling price. Competition is suggested to be promoted in the outpatient care market where hospitals have control over quality and government sets a limit on the prices that hospitals may charge.

**Supplementary Information:**

The online version contains supplementary material available at 10.1186/s13561-024-00516-4.

## Introduction

Quality improvement has become a central tenet in health care. The health care sector has traditionally been extensively regulated by governments in many countries to ensure the provision of high-quality healthcare services [1]. One of the most widespread examples is the public ownership of hospitals [2], as hospitals are essential providers of medical diagnostics and surgeries [3]. This has been the case in countries with a National Health System (such as the UK), the Scandinavian countries, and the southern European countries [1]. However, regulation and associated policies were sometimes found to be linked with adverse outcomes, pushing this sector further away from economic efficiency. For instance, public hospitals tended to overspend their budgets, while public-sector contractual arrangements were unable to solve moral hazard and adverse selection problems [2, 4]. This evidence encouraged some countries to enhance market mechanisms within their health systems [2]. Since the early 1990s, to enhance the quality of care and increase efficiency gains, governments in many countries launched a series of “top-down” pro-competition reforms [2, 5–19]. These reforms are commonly associated with mechanisms that impact both the supply [2, 5–18] and demand sides [2, 5, 13, 19] of the healthcare marketplace.

China has made substantial progress in widening the availability of healthcare services [20] and reducing the economic burden of many diseases over the last three decades [21]. Much of this improvement was attributed to unprecedented economic growth that lifted millions out of poverty [22] and an increase in government spending on public health [23]. But coinciding with the growth in incomes and health insurance coverage has been people’s demand for healthcare services and expectations for better quality of care [24]. Meanwhile, the overall healthcare market in China has been characterized for its “semi-control and semi-market” status [25, 26]. Under such a mixed system, problems can arise from excessive or inappropriate government intervention or too limited involvement [26, 27]. To elevate the quality of care and enhance efficiency of the healthcare system, the Chinese government initiated a series of pro-competition reforms from 2009 onwards [28, 29]. With the implementation of pro-competition policies [30, 31], hospital competition has intensified dramatically over the past decade in China [29]. As shown in Appendix 1, the total number of hospitals increased from 20,291 in 2009 (of which 22.39% were private hospitals) to 34,354 in 2020 (of which 65.27% were private hospitals) [32, 33]. Despite these efforts, it is still uncertain whether these pro-competitive reforms yielded desired health outcomes [28].

The impacts of hospital competition have been widely investigated in the literature from developed countries [5, 6, 14, 16, 34–48], but there is a paucity of evidence from developing countries [49–51]. The question of how hospital competition might affect the quality of care is a topic that has received considerable attention, but there is little consensus empirically [37, 52, 53]. While numerous studies have documented the effects of hospital competition on reducing mortality [1, 5, 14, 16, 51, 54, 55], readmissions [55], and length of stay [1, 56], the literature is not unanimous. Some studies have demonstrated that hospital competition was not associated with [40, 55, 57], or was inversely related to, the quality of care [44, 47, 48, 58]. Furthermore**,** most research has focused on how competition influenced the quality of inpatient care, while little is known about its impacts on the quality of outpatient care [51, 59]. Inpatients tend to exhibit much lower demand elasticities and place a greater relative valuation on distance versus quality when choosing a hospital [60]. This is because these individuals tend to choose nearby hospitals or ambulances usually take them to the closest hospital [37]. In comparison, there tends to be a higher demand elasticity for outpatient care [28]. This is because outpatients have the time and opportunity to substitute costly outpatient hospital care with less costly primary care or choose home-based self-treat options. Consequently, individuals who consider outpatient care are more likely to be attracted by the quality of care provided by hospitals. Additionally, the results from the literature on the relationship between competition and the quality of care varied by disease [42, 49]. Despite outpatient care constitutes the bulk of the health care, current literature attached more significance to acute diseases or surgeries. For instance, numerous related studies have dealt with acute illnesses or operations such as hip and knee replacements [5, 37, 44, 47], hip fracture [14, 42], stroke [14, 41, 42], and acute myocardial infarction [1, 14, 16, 41, 42, 58]. As such, this study assessed the impacts of hospital competition on the quality of outpatient care for patients with non-acute diseases in China.

## Methods

### Data sources

Our empirical analysis was guided by a theoretical model offered in Appendix 2. Patient-level data were extracted from the Municipal Human Resource and Social Security Bureau that links databases to patient-level data. The databases used in our research include demographic and outpatient encounter data at hospitals with public health insurance (PHI) coverage for patients covered by PHI programs in Changde city from 2015 to 2019^1^. In the databases, influenza-specific visits accounted for the highest market share across all disease categories, taking up more than 25% of the market over the study period (Appendix 3). Moreover, there has been a lack of research on the marketplace for the management for this condition wherein demand is anticipated to be highly elastic. For these reasons, this study included/focused on individuals with influenza. An unbalanced dataset comprising 406,664 individuals who had outpatient visits for influenza at hospitals^2^ from 2015 to 2019 was formed as our analysis sample. The steps by which our final analysis sample was obtained under specific exclusion criteria are detailed in Appendix 4.

Data on hospitals’ addresses were obtained from the online inquiry platforms of three companies that deliver basic registration information on enterprises in China (https://www.qc.com; https://www.qixin.com; https://www.qcc.com)^3^. We then manually collected the coordinates of hospitals using the “Baidu Coordinates Extraction System” (https://api.map.baidu.com/lbsapi/getpoint/index.html)^4^. To calculate the distance between hospitals, we extracted data on road networks and administrative boundary maps at the scale of 1:25,000 from the China National Earth System Science data center [61].

Data on the annual intensity of nighttime lights for hospitals were derived from the China Remote Sensing Satellite Ground Station of the Chinese Academy of Science (the “Flint” data) [62]. The “Flint” data were formed after processing the raw data recorded by the Visible Infrared Imaging Radiometer Suite sensor onboard the NPP satellite [63].

### Study variables

The quality of outpatient care was the outcome variable of interest. The occurrence of 14-day follow-up encounters for influenza could be regarded as a signal of poor quality of care 5[37]. Therefore, the occurrence of 14-day follow-up encounters for influenza at any healthcare facilities, outpatient facilities, and hospital outpatient departments were the three quality indicators used 6[64].

We included hospital competition as the key independent variable. We used the Herfindahl-Hirschman index (HHI) as a measure of competition as no consensus on preferred alternative measures, its good calculability, and its representability of both the number and the size of competitors [53]. As shown in Eq. 1, *X*_*j*_ and *X*_*m*_ represent the service volume supplied by hospital *j* and by *N* number of hospitals competing in the market *m* respectively. We assigned each hospital a unique market catchment area, which is the region enclosed by a circle centered on the hospital and is defined by a 5 km radius [65]. We also adopted alternative radii in our sensitivity analysis to test the robustness of our results with varying market radii. The value of the HHI ranges from 0 (representing perfect competition) to 1 (representing a monopoly), with a higher value indicating a lower level of hospital competition. The HHI was log-transformed to ease understanding and explanation.

1 $${HHI}_{j,m}=\sum_{j=1}^{N}{(\frac{{X}_{j,m}}{{X}_{m}})}^{2}$$

We included a set of covariates that have been shown to impact the quality of outpatient care. Theses variables included patient age (years) [66], gender (male and female) [46], whether the patient had comorbidities (whether the patient had other diseases than influenza) [42], and type of health insurance used to pay medical costs (Urban Employee Basic Medical Insurance, the UEBMI or Urban and Rural Resident Basic Medical Insurance, the URRBMI) [28]. A categorical variable was generated to measure the class of hospitals patients visited and we expected that higher-tier hospitals would provider better quality of outpatient care [67].

### Data analysis

Before estimating the impacts of hospital competition, we need to calculate the distance between hospitals and create a catchment area for each hospital. To ensure maximum accuracy, this study considered the actual road networks and travel times over difficult terrain when calculating the distance matrix. The Geographical Information-based approach has been widely adopted and effectively applied to carry out spatial accessibility of the healthcare services [68–70]. We performed the Origins-Destinations (OD) Cost Matrix analysis [71] to compute the distance matrix between hospitals. After that, we conducted the Service Area analysis [72] to create a 5/10/15 km distance-based catchment area for each hospital. The Buffer Analysis [73] and the Zonal Statistic analysis [74] were then conducted to derive the intensity of the nighttime lights for each hospital. The specific procedures of performing these spatial analyses were detailed in Appendix 5.

Considering that quality of outpatient care was measured by a binary variable, using the ordinary least square estimator is inappropriate given the inherent nonlinearity of the data. Instead, we adopted the nonlinear binomial logistic regression technique, the most common technique used to model binary data [75]. As we had a five-year longitudinal data, we constructed a mixed-effects model that allows for the examination of hypotheses concerning between-group differences in the mean structure [76]. Specifically, we fitted a three-way random intercepts model that adjusts time-, hospital-, and individual-level crossed random effects for each quality indicator. The regression model was described in Eq. 2: *Q*_*ijt*_ denotes the quality of outpatient care patient *i* received from hospital *j* in year *t*; *β*_*1*_ represents the coefficient on the HHI in the determination of the quality of outpatient care; ${\beta }_{2}$ represents a vector of the effects of individual-level covariates.

2 $${Q}_{i,j,t}={\alpha }_{i,j}+ {\beta }_{1}{HHI}_{j,t}+{\beta }_{2}X+{\vartheta }_{i,j,t}\, with \,{\alpha }_{i,j}={\alpha }_{i}+{\gamma }_{i,j} ,{\alpha }_{i}={\alpha }_{0}+{\omega }_{i}$$

The endogeneity concern of the HHI is a major problem that needs to be solved when estimating its impacts on the quality of outpatient care [77]. A standard method adopted by previous literature to address such endogeneity concern was to calculate the HHI based on the predicted patient choices [47]. We were unable to employ such a method as more than half of our sampled patients had missing values for their addresses^7^. We tried to create a dummy distance variable (whether patients visited hospitals in their own county/district) based on their identity number provided in the dataset^8^. Unfortunately, the results of the conditional logit regression model suggest that the categorical distance variable had no statistically significant impact on patients’ choices of hospitals. One possible explanation of this result is that patients with influenza were unlikely to seek outpatient care outside their own county/district. As such, we considered external IVs.

We considered nighttime nights as a valid instrument variable primarily for two reasons, including instrumental relevance and exclusion restriction. Prior research has shown that nighttime lights can capture some important dimensions of human development, including access to healthcare services [78]; hence, nighttime lights would be correlated with hospitals’ total service volume and the associated measure of hospital competition. Meanwhile, nighttime lights would not affect the quality of outpatient care received by outpatients as these individuals would not stay at hospitals overnight. We also conducted a review to check whether any prior studies could provide support for the association between nighttime lights and the quality of outpatient care. Searches were undertaken in PubMed, JSTOR, and Google Scholar using a combination of the key concepts (nighttime light and quality) with appropriate truncations and wildcards. This searching process led to a total of 56 articles, from which we did not find any evidence suggesting that nighttime light was associated with the quality of outpatient care. We thus used the intensity of nighttime lights (for hospital *j* in year *t*)^9^ as an IV for hospital demand (for hospital *j* in year *t*). Subsequently, the fitted demand was used to calculate the value of the market share and the associated competition measure (the HHI) for each hospital.

To test the robustness of our research findings, we conducted three sensitivity analyses. First, we used whether the patient had 14-day follow-up encounters to the same hospital outpatient department as a measure of the quality of outpatient care. Second, we instead used different market radii to examine whether the impacts of the HHI were sensitive to market size. Third, we estimated the impacts of the HHI on the quality of outpatient care measured by whether the patient had 7-day follow-up encounters for influenza. Finally, we performed heterogeneity analysis for different factors, including age^10^, type of health insurance, hospital class, and comorbidity status. All the spatial analyses were conducted using the Network Analyst tool and the Spatial Statistics toolbox in the ArcGIS Desktop 10 [79]. All the econometric analyses were performed using the open-source programming language R [80].

## Results

### The descriptive results of the sampled outpatients

Appendix 6 shows changes in the annual mean value of the HHI for all the sampled hospitals from 2015 to 2019. The mean value of the HHI fell over the study period for all the HHI measures based on different market radii, indicating the presence of a more competitive market. As expected, the value of the HHI decreased with market radius as more and more hospitals were included in the set of competitors from which the HHI was measured. Appendix 7 shows changes in the annual mean quality level of outpatient care over the study period. The percentage of individuals who had 14-day follow-up encounters for influenza increased from 2015 to 2019, implying an overall downward trend in the quality of outpatient care.

Table 1 describes the descriptive characteristics of the research participants. A total of 406,664 outpatients were included for analysis. Most of the sampled outpatients were male (52.41%), used the UEBMI to pay their medical costs (88.11%), did not have comorbidities (59.78%), and visited the second-class hospitals (67.82%). The percentage of the sampled outpatients who had 14-day follow-up encounters for influenza at any healthcare facilities, outpatient facilities, and hospital outpatient departments was 38.29%, 6.04%, and 5.06%, respectively.

**Table 1:** The descriptive characteristics of the sample outpatients

Variables	Unit	Min	Max	Med	Mean	SD
***Dependent variables***						
14-day follow-up encounters at any healthcare facilities	Binary	No = 250,972 (61.715%)				
		Yes = 155,692 (38.285%)				
14-day follow-up encounters at any outpatient facilities	Binary	No = 382,113 (93.963%)				
		Yes = 24,551 (6.037%)				
14-day follow-up encounters at any hospital outpatient departments	Binary	No = 386,077 (94.938%)				
		Yes = 20,587 (5.062%)				
***Independent variables***						
HHI (5 km)	Number	0.154	1.000	0.489	0.492	0.207
HHI (10 km)	Number	0.151	1.000	0.495	0.46	0.198
HHI (15 km)	Number	0.115	1.000	0.466	0.421	0.204
***Control Variables***						
Age	Number	1	103	53	52.809	15.709
Gender	Binary	Female = 193,537 (47.591%);				
		Male = 213,127(52.409%)				
Comorbidity	Binary	No = 243,086 (59.776%);				
		Yes = 163,578 (40.224%)				
Insurance	Categorical	Working residents with the URRBMI = 26,270 (6.460%);				
		Non-working residents with the URRBMI = 22,081 (5.430%);				
		Working employees with the UEBMI= 217,720 (53.538%)				
		Non-working employees with the UEBMI= 140,593 (34.572%)				
Hospital class	Categorical	Non-accredited = 20,748 (5.102%);				
		Accredited first-class = 104,091 (25.596%):				
		Accredited second-class = 275,806 (67.822%);				
		Accredited third-class = 6,019 (1.480%)				

Min, Max, and Med represent the minimum, maximum, and median value of the study variable, respectively

### The impacts of hospital competition on the quality of outpatient care

Table 2 reports the impacts of the HHI on the odds of having follow-up encounters at a range of healthcare settings (without using the IV approach). The estimated odds ratio (OR) of the HHI in predicting the probability of having 14-day follow-up encounters for influenza at any healthcare facilities, outpatient facilities, and hospital outpatient departments was 1.35 (*p* < 0.001), 1.18 (*p* < 0.001), and 1.21 (*p* < 0.001), respectively. These results imply that for each 1% increase in the degree of hospital competition, an individual’s odds of having 14-day follow-up encounters for influenza at any healthcare facilities, outpatient facilities, and hospital outpatient departments fell by 34.9%, 18.3%, and 20.9%, respectively^11^.

**Table 2:** The impact of competition on the odds of having follow-up encounters (without IV)

	Any healthcare facilities				Any outpatient facilities				Any hospital outpatient departments			
	Estimate	SE	*P*-value	Sig.	Estimate	SE	*P*-value	Sig.	Estimate	SE	*P*-value	Sig.
Intercept	10.621	0.501	< 0.001	***	-9.82	0.416	< 0.001	***	-10.135	0.411	< 0.001	***
Log(HHI)	0.299	0.042	< 0.001	***	0.168	0.035	< 0.001	***	0.189	0.035	< 0.001	***
Log(age)	0.316	0.128	0.014	*	-0.059	0.095	0.535		-0.016	0.096	0.87	
Gender_female												
Gender_male	0.135	0.061	0.027	*	0.048	0.045	0.285		0.04	0.045	0.37	
Insurance_working employees												
Insurance_working residents	-25.505	0.179	< 0.001	***	-23.217	0.157	< 0.001	***	-23.223	0.159	< 0.001	***
Insurance_non-working residents	-26.422	0.245	< 0.001	***	-24.701	0.236	< 0.001	***	-24.756	0.235	< 0.001	***
Insurance_non-working employees	-0.194	0.085	0.022	*	-20.978	0.087	< 0.001	***	-21.022	0.082	< 0.001	***
Comorbidity_no												
Comorbidity_yes	-0.215	0.058	< 0.001	***	-0.129	0.039	0.001	***	-0.131	0.039	0.001	***
Class_non-accredited												
Class_first	1.283	0.147	< 0.001	***	21.377	0.218	< 0.001	***	21.395	0.219	< 0.001	***
Class_second	1.204	0.148	< 0.001	***	21.57	0.22	< 0.001	***	21.622	0.221	< 0.001	***
Class_third	0.99	0.524	0.059	.	21.328	0.531	< 0.001	***	21.31	0.536	< 0.001	***
***Random intercepts***												
Time	Yes											
Hospital	Yes											
Individual	Yes											

SE represents the standard error; Sig. represents the significance codes: < 0.001 ***, < 0.01**, < 0.05 *, < 0.01

Table 3 reports the results of the impacts of the HHI on the odds of having follow-up encounters at a range of healthcare settings (using the IV approach). The results of the first-stage regression suggest that the intensity of the nighttime lights was positively associated with hospital demand (*p* < 0.01). The p-value of the Wald Chi-Squared test was less than 0.01, rejecting the presence of weak instruments. These results justify the appropriateness of using the intensity of nighttime lights as an IV in our study. The estimated OR of the HHI in predicting the probability of having 14-day follow-up encounters for influenza at any healthcare facilities, outpatient facilities, and hospital outpatient departments was 1.29 (*p* < 0.001), 1.12 (*p* < 0.001), and 1.12 (*p* < 0.001), respectively. These findings imply that for each 1% increase in the degree of hospital competition, the odds that a patient had 14-day follow-up encounters for influenza at any healthcare facilities, outpatient facilities, and hospital outpatient departments fell by 28.6%, 12.2%, and 12.3%, respectively.

**Table 3:** The impacts of competition on the odds of having follow-up encounters (with IV)

	Any healthcare facilities				Any outpatient facilities				Any hospital outpatient departments			
	Estimate	SE	*P*-value	Sig.	Estimate	SE	*P*-value	Sig.	Estimate	SE	*P*-value	Sig.
Intercept	10.888	0.503	< 0.001	***	-9.741	0.416	< 0.001	***	-9.882	0.419	< 0.001	***
Log(HHI)	0.252	0.03	< 0.001	***	0.115	0.025	< 0.001	***	0.116	0.024	< 0.001	***
Log(age)	0.299	0.128	0.019	*	-0.062	0.094	0.51		-0.044	0.095	0.641	
Gender_female												
Gender_male	0.128	0.061	0.036	*	0.048	0.045	0.288		0.041	0.045	0.366	
Insurance_working employees												
Insurance_working residents	-25.668	0.183	< 0.001	***	-23.256	0.158	< 0.001	***	-23.297	0.161	< 0.001	***
Insurance_non-working residents	-26.47	0.244	< 0.001	***	-24.716	0.236	< 0.001	***	-24.703	0.235	< 0.001	***
Insurance_non-working employees	-0.192	0.085	0.023	*	-20.977	0.084	< 0.001	***	-21.003	0.083	< 0.001	***
Comorbidity_no												
Comorbidity_yes	-0.202	0.059	0.001	***	-0.128	0.039	0.001	***	-0.136	0.039	< 0.001	***
Class_non-accredited												
Class_first	1.232	0.147	< 0.001	***	21.357	0.219	< 0.001	***	21.425	0.219	< 0.001	***
Class_second	1.178	0.146	< 0.001	***	21.567	0.22	< 0.001	***	21.669	0.22	< 0.001	***
Class_third	1.028	0.524	0.05	*	21.327	0.532	< 0.001	***	21.355	0.534	< 0.001	***
***Random intercepts***												
Time	Yes											
Hospital	Yes											
Individual	Yes											

SE represents the standard error; Sig. represents the significance codes: < 0.001 ***, < 0.01**, < 0.05 *, < 0.01

In all the models, the impacts of age, health insurance status, comorbidity status, and hospital class on the quality of outpatient care were consistent. Older individuals and males were more likely to have 14-day follow-up encounters for influenza at any healthcare facilities than their counterparts. Patients who visited the accredited hospitals were more likely to have 14-day follow-up encounters for influenza than their counterparts. Individuals who used the URRBMI to pay their medical costs were less likely to have 14-day follow-up encounters for influenza than those whose medical costs were reimbursed by the UEBMI.

### The results of sensitivity analyses

Table 4 reports the impacts of the HHI on whether the patient had 14-day follow-up encounters for influenza at the same hospital outpatient department. We found that hospital competition resulted in a reduction in the probability of having 14-day follow-up encounters at the same hospital outpatient department. The impacts of the HHI measured based on different market radii were reported in Table 5. Hospital competition was still found to improve the quality of outpatient care when the market radius was set to 5 km and 10 km. The results of heterogeneity analysis were visualized in Appendix 8, Appendix 9, and Appendix 10. We found that an increase in hospital competition was associated with a decline in the quality of outpatient care among those who visited non-accredited hospitals. In contrast, females, adults aged 25 to 64 years, those using the URRBMI to pay medical expenses, and those with comorbidities tended to benefit from the positive health effects of hospital competition than their counterparts. These results demonstrated that our findings concerning the effects of hospital competition on increasing the quality of outpatient care from the primary analysis were robust.

**Table 4:** The impacts of competition on the odds of having follow-up encounters at the same hospital outpatient department

	OLS				IV			
	Estimate	SE	*P*-value	Sig.	Estimate	SE	*P*-value	Sig.
Intercept	-9.899	0.416	< 0.001	***	10.168	0.399	< 0.001	***
Log(HHI)	0.184	0.035	< 0.001	***	0.092	0.025	< 0.001	***
Log(age)	-0.045	0.095	0.634		-0.049	0.097	0.61	
Gender_female								
Gender_male	0.035	0.045	0.436		0.039	0.045	0.388	
Insurance_working employees								
Insurance_working residents	-23.251	0.16	< 0.001	***	-23.627	0.167	< 0.001	***
Insurance_non-working residents	-24.671	0.235	< 0.001	***	-24.69	0.233	< 0.001	***
Insurance_non-working employees	-21.029	0.084	< 0.001	***	-20.935	0.084	< 0.001	***
Comorbidity_no								
Comorbidity_yes	-0.144	0.039	< 0.001	***	-0.12	0.039	0.002	**
Class_non-accredited								
Class_first	21.397	0.218	< 0.001	***	1.149	0.164	< 0.001	***
Class_second	21.638	0.221	< 0.001	***	1.407	0.165	< 0.001	***
Class_third	21.312	0.535	< 0.001	***	1.03	0.514	0.045	*
***Random intercepts***								
Time	Yes							
Hospital	Yes							
Individual	Yes							

SE represents the standard error; Sig. represents the significance codes: < 0.001 ***, < 0.01**, < 0.05 *, < 0.01

**Table 5:** The impacts of competition with different market radii on the odds of having follow-up encounters

	Model A		Model B		Model C	
	10 km					
	Coeff.	Sig.	Coeff.	Sig.	Coeff.	Sig.
Intercept	-8.644 (0.508)	***	10.567 (0.404)	***	-9.765 (0.421)	***
Log(HHI)	0.275 (0.028)	***	0.094 (0.023)	***	0.125 (0.023)	***
Log(age)	0.301 (0.123)	*	-0.094 (0.096)		-0.049 (0.095)	
Gender_male	0.141 (0.061)	*	0.048 (0.045)		0.038 (0.045)	
Insurance_working residents	-25.108 (0.177)	***	-23.66 (0.165)	***	-23.341 (0.162)	***
Insurance_non-working residents	-26.362 (0.243)	***	-24.659 (0.235)	***	-24.698 (0.235)	***
Insurance_non-working employees	-0.177 (0.083)	*	-20.855 (0.084)	***	-20.999 (0.084)	***
Comorbidity_yes	-0.209 (0.058)	***	-0.108 (0.039)	**	-0.134 (0.039)	***
Class_first	20.786 (0.218)	***	1.168 (0.161)	***	21.382 (0.219)	***
Class_second	20.742 (0.217)	***	1.353 (0.163)	***	21.61 (0.221)	***
Class_third	20.748 (0.544)	***	1.137 (0.507)	*	21.368 (0.533)	***
	15 km					
	Coeff.	Sig.	Coeff.	Sig.	Coeff.	Sig.
Intercept	10.875 (0.502)	***	10.163 (0.398)	***	10.099 (0.401)	***
Log(HHI)	0.205 (0.026)	***	0.057 (0.022)	**	0.057 (0.022)	**
Log(age)	0.295 (0.128)	*	-0.06 (0.096)		-0.044 (0.097)	
Gender_male	0.121 (0.061)	*	0.054 (0.045)		0.046 (0.045)	
Insurance_working residents	-25.668 (0.164)	***	-23.565 (0.165)	***	-23.617 (0.168)	***
Insurance_non-working residents	-26.489 (0.23)	***	-24.751 (0.234)	***	-24.724 (0.233)	***
Insurance_non-working employees	-0.188 (0.085)	*	-20.882 (0.084)	***	-20.909 (0.084)	***
Comorbidity_yes	-0.195 (0.059)	***	-0.103 (0.039)	**	-0.113 (0.039)	**
Class_first	1.279 (0.146)	***	1.197 (0.161)	***	1.178 (0.164)	***
Class_second	1.223 (0.146)	***	1.408 (0.162)	***	1.424 (0.165)	***
Class_third	1.092 (0.523)	*	1.111 (0.509)	*	1.05 (0.513)	*

Models A, B and C represent the model where any healthcare facilities, any outpatient facilities, any hospital outpatient departmentswas included as the dependent variable; Standard errors in parentheses; Sig. represents the significance codes: < 0.001 ***, < 0.01**, < 0.05 *, < 0.01 .; The individual-, hospital-, and time-level random intercepts were included in all the model

## Discussion

This study constructed a binomial regression model with cross random intercepts and employed an IV approach to estimate the health effects of hospital competition. Our research found that after adjusting for various covariates, hospital competition improved the quality of outpatient care for those with influenza in China. The health benefits of hospital competition were more substantial among females, those using the URRBMI to pay for medical costs, those visiting the accredited hospitals, and those aged 25 to 64 years when compared with their counterparts.

Our study demonstrated that hospital competition increased the quality of outpatient care. While this finding is consistent with some prior studies [1, 5, 14, 16, 51, 54–56], it is at odds with others [40, 44, 47, 48, 55, 57, 58]. Competition generally was linked with quality improvements, though this may not be the case when hospitals exhibit altruistic motivations [1, 6, 42, 44, 81], face capacity constraints [82], have a limited ability to appropriate their profits [44, 83, 84], and are able to compete on both quality and prices [52, 85]. As suggested by our theoretical analysis offered in Appendix 2, competition may lead to poorer quality when the quality elasticity of demand is smaller than the doubled price elasticity of demand. This assumption is unlikely to be true in our empirical setting, as all the sampled hospitals had PHI coverage and were subject to the associated price regulations. Accordingly, instead of competing on price, these sampled hospitals would prefer to provide higher quality services to gain an advantage over their rivals. In a summary, it was expected that the demand for outpatient care would respond more elastically to quality changes than to price variations in China. This may explain why an increase in hospital competition was associated with better quality of outpatient care for individuals with influenza in our study.

We also demonstrated that age, gender, health insurance status, and hospital class were associated with the quality of outpatient care, which aligns with prior studies [42, 46, 51, 66, 86, 87]. Specifically, we found that patients who visited the accredited hospitals tended to experience poorer quality of outpatient care than their counterparts. Hospitals in China are classified into three tiers based on hospitals’ capacity to provide healthcare services, medical education, and conduct medical research. However, a hierarchical diagnostic and treatment system has not yet been established across China, and patients still have the freedom to choose hospitals they prefer. These may account for why patients visiting the accredited hospitals achieved poorer health outcomes when compared with those who visited the non-accredited hospitals. One unexpected finding is that patients with comorbidities experienced lower quality of outpatient care than their counterparts. Our focus on influenza-specific quality indicators may offer explanations for this finding as influenza may not be the most responsible diagnosis for patients with comorbidities.

Our heterogeneity analysis indicated that the effects of hospital competition on improving the quality of outpatient care were more substantial among individuals who used the URRBMI to pay their medical expenses, females, adults aged 25 to 64 years, and those with comorbidities when compared with their counterparts. In contrast, an increase in hospital competition was found to reduce the quality of outpatient care among patients who visited the non-accredited hospitals. Non-accredited hospitals often faced challenges in delivering high-quality outpatient care due to limited capacity in promoting innovation. As a result, these hospitals may prioritize patient retention as a strategic response rather than referring patients to other healthcare facilities when confronted with intensified competition.

Some policy suggestions can be derived from this study. We demonstrated that hospital competition enhanced the quality of outpatient care. As such, policy decision-makers are suggested to further promote competition among hospitals in the outpatient care market in China. An official information-sharing platform [14, 54] is suggested to be established to report the quality of care offered by hospitals. A performance-based financial mechanism [16] and a prospective payment system [88, 89] are suggested to be constructed simultaneously.

Several strengths of this study should be acknowledged in interpreting our findings. While prior studies have assessed the impacts of hospital competition in developed countries, we contributed to the literature by offering evidence from developing countries. Additionally, much of previous studies have focused on the health effects of hospital competition under a fixed-price regime. Our study contributed to extant research by adding evidence on potential health gains from increasing hospital competition under price-cap regulation. Third, we used the Geographical Information-based techniques to calculate the actual road distance between hospitals, which greatly improved the accuracy of the estimates. Finally, research that adopted a cross-sectional design or did not address the endogeneity of the HHI was unable to reveal the causal relationship [37]. Our study overcame this shortcoming by using a large longitudinal dataset and adopting an IV approach to estimate the causal impacts of the HHI.

Our study exhibited several limitations. Our sample data were restricted to patients with PHI coverage who had outpatient visits at hospitals that opt-in to the PHI arrangements from 2015 to 2019. We were thus unable to investigate how hospital competition might affect health for all the patients regardless of whether being covered by PHI programs. Notwithstanding this limitation, PHI coverage was prevalent among citizens in Changde during our study period. Accordingly, our findings should be generally applicable to most citizens of Changde. Second, we adopted utilization-based indicators as a proxy for the quality of outpatient care due to their widespread application in the extant literature and availability of data. There were two potential concerns associated with the use of the readmission measure, which could introduce biases to our estimation results: 1) the quality of care received at primary care facilities after leaving a hospital may impact the odds of having a follow-up visit; and 2) the occurrence of having a follow-up visit to a healthcare facility within medical alliances may not necessarily indicate poor quality of care received. In many countries, primary care serves as the initial point of contact for individuals seeking healthcare services and plays a fundamental role in facilitating follow-up care [90]. However, in China, despite significant government investments in developing primary care, patients often lack trust in primary care and tend to bypass primary facilities to visit hospitals for any health issues [91]. Additionally, we were unable to access information on which institutions implemented the medical alliance modes and whether a follow-up visit represents a referral between two facilities within the medical alliances. In the absence of mandatory implementations of a hierarchical diagnosis and treatment system and a medical alliance mode, we expected that biases associated with the readmission measure would exist only to a limited extent. Nevertheless, we only used one proxy for quality of outpatient care, which may lead to a partial picture of the impacts of hospital competition [6]; we hence highly recommend future research with a more detailed tracking of patients to generate a comprehensive and in-depth understanding of the health effects of hospital competition. Third, as it was hard to find instruments for the service volume of non-hospital facilities, we excluded the service volume of these facilities when calculating the HHI for each hospital. This resulted in a larger market share and a smaller HHI value for each hospital, which may bias the estimation of the impacts of the HHI. Prior research has shown a downward pressure on revenues, costs, and profits in general hospitals associated with the presence of other types of healthcare facilities [92]. We therefore strongly recommend future research to estimate the impacts of competition between hospitals and other non-hospital facilities. Fourth, a hospital is a multi-product firm in which competition in one product area may have spillover effects on the quality of other products [47]. We did not consider such effect because of the rare occurrence of inpatient visits for influenza and the large variances in the market share of other diseases in our dataset. Future research is suggested to investigate the spillover effects of competition when estimating the overall health effects of hospital competition. Finally, due to data unavailability, this study was unable to explore the underlying mechanisms through which the positive effects of hospital competition occur. This may open another potentially fruitful line of investigation into the health effects of hospital competition.

## Conclusions

Our study demonstrated a temporal trend towards increased competition among hospitals, and surprisingly, an associated decline in the quality of outpatient care. When the effects of competition were assessed among patients and over the whole study period, hospital competition enhanced the quality of outpatient care. The evidence offered herein of the positive impacts of hospital competition suggests that pro-competition efforts may yield dividends in the outpatient market.

### Supplementary Information

Supplementary Material 1.

## Data Availability

No datasets were generated or analysed during the current study.

## Abbreviations

HHI: Herfindahl-Hirschman index
PHI: Public health insurance
UEBMI: Urban Employee Basic Medical Insurance
URRBMI: Urban and Rural Resident Basic Medical Insurance
OD: Origins-Destinations
OR: Odds ratio

## Footnotes

1 More than 97% of citizens in Changde city were covered by PHI programs by the end of 2019 (https://zwgk.changde.gov.cn/public/6617368/8285887.html).

2 This indicates that only hospitals with at least one encounter in the dataset were included in this study. Since we had a five-year longitudinal dataset, we expected our dataset to cover most of the hospitals that opt-in to the PHI arrangements in Changde city.

3 We assigned different coordinates to hospitals that have multiple locations (2 out of 141 hospitals). Hospitals included in this study had separate financial and personnel management systems. Hospitals that opt-in to the PHI arrangements are required to comply with a set of regulations on their marketing behaviors. As such, hospitals included in this study were assumed to operate independently and we did not consider the possibility that they may adopt common strategic plans.

4 We manually collected the coordinates of all the hospitals as some hospitals could not be accurately located when using the programming software to convert their addresses to geographic coordinates.

5 We tried to use the number of emergency visits as a proxy for the quality of outpatient care. Nevertheless, we found that only 6 out of 406,664 sampled individuals had all-cause emergency visits, so we dropped this quality indicator.

6 For sampled individuals who had outpatient visits in the last month of 2019, data in January 2020 were additionally collected and used to derive the quality indicators.

7 This is because that our original dataset was used by the government to reimburse individuals. Reimbursements were invariant to individuals’ residence and therefore data on individuals’ residence were not collected by the government.

8 The identity number is usually 15-19 digits long, with number 5-6 representing the county/district in which the patient lives. There was no missing value on individuals’ identity number in our dataset.

9 The intensity of nighttime lights was measured by a hospital’s total luminance of night-time lights divided by the sum of the number of the pixels in the imageries. Each pixel has a digital number value ranging from 0 to 63, with higher digital number values indicating more intense lights.

10 Individuals were grouped into four age categories: children aged between 0 to 14 years, youth aged between 15 to 24 years, adults aged between 25 to 64 years, and seniors aged between 65 years and older.

11 We made such explanation as: 1) the logarithm used to transform our independent variable is the same as the base used for the log of OR in the logistic regression; and 2) the estimated regression coefficients are close to 0.
