# Supplementary material for: Does hospital competition improve the quality of outpatient care? - empirical evidence from a quasi-experiment in a Chinese city
Source: Health Econ Rev. 2024 Jun 8;14:39. doi: 10.1186/s13561-024-00516-4 (PMC11162028; doi:10.1186/s13561-024-00516-4)
Supplement: Supplementary file 1 — Supplementary Material 1. [file 13561_2024_516_MOESM1_ESM.docx]

**Appendix 1 The number of hospitals in China from 2010 to 2019**

**Note:** The figure was made by the author.

**Appendix 2** **The theoretical model**

Competition is a key concept in economics: competitive markets allow economies to efficiently allocate resources via a system of decentralized decisions [1]. Crucial for the working of this mechanism is the effect of incentives provided by price [1], as price is one of the determining factors for purchasing decisions by consumers. In China, public health insurance (hereafter referred to as PHI) program consists of the following key schemes: 1) the Urban Employee Basic Medical Insurance (UEBMI) for the urban employed individuals; 2) the URRBMI covering rural and urban residents without formal employment^1^; and 3) other public health insurance programs, such as programs that target the poor and disadvantageous groups. Hospitals covered by PHI programs comprise all public hospitals as well as a set of private hospitals that have had their services authorized for coverage. Under the PHI arrangements, the covered hospitals are required to comply with a single common maximum list of prices for each type of product set by the government. These products comprise healthcare services and drugs that are included under the China National Basic Medical Insurance, Work Injury Insurance, and Fertility Insurance Drug Catalogue, the Diagnosis and Treatment Items Catalogue, and the Medical Services Facilities Catalogue (“Insurance Catalogue”). In contrast, all other private hospitals must completely opt-out of PHI coverage but have autonomy to set their own prices for healthcare services. While the price of some hospital products (e.g., medical diagnosis and drugs) are regulated in China, some are left to be determined by the market. Additionally, the total charge faced by patients for their episode of care is not fixed; hence, there may still exist potential price competition between hospitals in China [2]. In particular, hospitals may lower the total price of treatments to attract patients who are price sensitive.

The second essential dimension through which hospitals compete for patients is to raise the quality of care they offer. Under a regime with a price-cap regulation, competition is manifest through both price and quality dimensions but there are limits to price competition when hospitals agree to be covered by PHI programs [1, 3]. Third, some prior studies have shown that hospitals may exhibit altruistic preferences; accordingly, these hospitals may trade-off benefits to patients against lower profits [4, 5].

Based on the above considerations, this paper considers a hospital’s optimization problem as a utility maximization problem. A hospital’s utility was assumed to depend on two linearly additive components (as described in Equation 1): 1) profit, $\pi$, represented as total revenues minus total sales costs; and 2) an altruistic component, $\propto\times B \mathrm{with}\propto\in(0,1)$, that captures a hospital’s non-monetary altruistic motive to promote quality (*q*) while lowering prices (*p*) it charges to consumers. Particularly, a set of regulated ceiling prices that varied by type of product were applied to hospitals that were under the regulatory arrangements of the PHI either because they were public hospitals or because they were private hospitals that had their products authorized for the PHI coverage. Consequently, the optimization problem faced by a hospital is to maximize utility by setting prices, *p*, and the quality of care, *q*, subject to an upper regulatory limit to prices, *p^r^*:

$$(1) Maximize U_{hospital}$$

$$=\pi\left( p,q; \theta\right)+\propto\times B\left( p,q \right)$$

$$=p\times D\left( p,q;\theta\right)-C[D\left( p,q; \theta\right),q]+\propto\times B(p,q)$$

$$subject to p^{r}- p\geq0$$

Where:

*D (p,q;θ*) represents the total demand for treatments at a hospital. *D* is modelled as a decreasing function of price (*D_p_ < 0*), an increasing function of quality (*D_q_ > 0)*, and a decreasing function of market competition (*D_θ_ < 0*). Market competition was assumed to change the responsiveness of demand to quality and price. Crucially, we assumed that competition mitigates the negative demand elasticity of price (*D_pθ_ < 0*) while increasing the positive demand elasticity of quality (*D_qθ_ > 0*) [5].

*C* represents the total costs of supplying hospital treatments, which is determined by hospital demand (*C_D_* > *0*) and quality of hospital treatments *(C_q_ > 0)*. We allowed for cost substitutability (*C_Dq_ > 0*) and complementarity (*C_Dq_ < 0*) between demand and quality. Cost substitutability (complementarity) means that the marginal cost of supplying quality is increasing (decreasing) in demand.

*B* represents the total non-monetary utility of patients receiving treatments at a hospital, which is modelled as a function of quality (*B_q_ > 0*) [6] and price (*B_p_ < 0*). We did not differentiate private hospitals from public ones as we assumed that both private and public hospitals, at least to some degree, would behave altruistically [6, 7].

The first order conditions with respect to price, *p*, and quality, *q*, (*p > 0* and *q > 0*) were given in Equations 2 to 3. The left-hand side of Equation 2 represents the marginal cost of providing an additional hospital product. The right-hand side of Equation 2 represents the marginal utility of providing an additional hospital product: 1) the first term denotes the regulated price plus the shadow price of the price margin (between the retail price and the regulated price) as a percent of the price elasticity of demand; 2) the second term denotes the percentage of a hospital’s demand as a percent of the price elasticity of demand; and 3) the third term represents the price elasticity of altruistic benefit as a percent of the price elasticity of demand. The left-hand side of Equation 3 represents the marginal cost of providing quality. The right-hand side of Equation 3 represents the marginal utility that a hospital can derive from providing quality: 1) the first term denotes the marginal monetary utility of providing quality, which is proportional to the gap between the regulated price and the marginal cost of providing an additional hospital product; and 2) the second term represents the marginal altruistic benefit of providing quality.

$$(2) C_{D}=(p^{r}+\frac{\lambda}{D_{p}})+\frac{D}{D_{p}}+\frac{\propto B_{p}}{D_{p}}$$

$$(3) C_{q}=(p^{r}-C_{D})\times D_{q}+{\propto B}_{q}$$

Totally differentiating the first order conditions and applying Cramer’s rule give the effects of competition (*θ*) on the equilibrium quality (*q**). We found that the association between competition and quality is ambiguous. To obtain intuition for the elasticity of interest, we made some strong simplifying assumptions following the spirit of prior work [5]: 1) constant price and quality elasticity of demand (*D_PP_ = 0* and *D_qq_ = 0*); 2) constant price and quality elasticity of cost (*C_qq_=0* and *C_DD_ = 0*); 3) constant price and quality elasticity of altruistic benefit (*B_pp_ =0* and *B_qq_ =0*); and 4) the marginal demand, cost, and altruistic benefit of price are invariant to quality variations (*D_Pq_ = 0*, *C_qD_ = 0*, and *B_pq_ = 0*). Based on the above assumptions, the impacts of competition ($\theta$) on the equilibrium quality ($q^{*}$) were given as follows:

$$(4) \frac{\partial q^{*}}{\partial\theta}=\underset{>0}{\underbrace{-\frac{D_{\theta}}{D_{q}}}}-\underset{>0}{\underbrace{\frac{(p^{r}-C_{D})}{D_{q}D_{q}}}}(\underset{<0}{\underbrace{D_{p\theta}D_{q}}}-\underset{<0}{\underbrace{D_{q\theta}2D_{p}}})$$

Equation 4 implies that the effects of competition depend on the marginal demand of price (*D_p_*) and quality (*D_q_*), and the impacts of competition on the marginal demand of price ($D_{p\theta}$) and quality ($D_{q\theta}$). Intuitively, if 1) the negative effects of competition on the price elasticity of demand can be offset by the positive effects of competition on the quality elasticity of demand ($D_{q\theta}>|D_{p\theta}|$), and 2) the quality elasticity of demand is smaller than the doubled price elasticity of demand ($|2D_{p}$| > $D_{q}$), the hospital will make a loss on marginal patients as $-(p^{r}-C_{D})<0$. In this case, the hospital will reduce quality since competition will increase the marginal loss from an increase in quality. Allowing for cost substitutability/complementarity and inefficiency in medical spending (relaxing *C_qD_ = 0* and *C_qq_ =* 0) offers consistent conclusions regarding the effects of competition*.*

**Appendix** **3 The diseases ranking top five in market share in Changde city from 2015 to 2019**

| **Year** | **Diseases** | **Number of visits** | **Market share** |
| --- | --- | --- | --- |
| 2015 | Influenza | 239,191 | 38.84% |
|  | Influenza (virus identified) | 65,655 | 10.66% |
|  | Coronary heart disease | 2,867 | 0.47% |
|  | Viral conjunctivitis | 1,985 | 0.32% |
|  | Influenza pneumonia | 1,792 | 0.29% |
| 2016 | Influenza | 246,138 | 36.51% |
|  | Influenza (virus identified) | 75,235 | 11.16% |
|  | Viral conjunctivitis | 3,280 | 0.49% |
|  | Coronary heart disease | 2,610 | 0.39% |
|  | Upper respiratory infection | 1,603 | 0.24% |
| 2017 | Influenza | 507,104 | 30.43% |
|  | Influenza (virus identified) | 30,053 | 1.80% |
|  | Upper respiratory infection | 15,489 | 0.93% |
|  | Schistosomiasis | 13,089 | 0.79% |
|  | High blood pressure | 12,257 | 0.74% |
| 2018 | Influenza | 566,833 | 26.22% |
|  | High blood pressure | 32,521 | 1.50% |
|  | Upper respiratory infection | 25,543 | 1.18% |
|  | Coronary heart disease | 16,217 | 0.75% |
|  | Chronic gastritis | 13,888 | 0.64% |
| 2019 | Influenza | 644,788 | 25.73% |
|  | High blood pressure | 47,571 | 1.90% |
|  | Upper respiratory infection | 29,783 | 1.19% |
|  | Bronchitis | 16,761 | 0.67% |
|  | Coronary heart disease | 16,483 | 0.66% |

**Note:** The market share was measured based on the number of outpatient visits in our datasets.

**Appendix** **4 The exclusion criteria of the research sample**

| ***Steps*** | ***Exclusion criteria*** | ***Number of individuals (institutions)*** | ***Number by year*** | | | | |
| --- | --- | --- | --- | --- | --- | --- | --- |
|  |  |  | ***Year 2015*** | ***Year 2016*** | ***Year 2017*** | ***Year 2018*** | ***Year 2019*** |
| 1 | All individuals | 11,302,026  (2,477) | 1,749,047  (1,231) | 1,799,637  (1,348) | 2,470,523  (2,048) | 2,534,976  (2,244) | 2,747,879  (2,347) |
| 2 | Excluding individuals without influenza | 7,967,086  (2,069) | 1,478,042  (1,202) | 1,507,251  (1,318) | 2,470,523  (1,686) | 1,608,939  (1,772) | 1,722,206  (1,849) |
| 3 | Excluding individuals who did not have outpatient visits for influenza | 976,375  (651) | 109,572  (254) | 108,049  (263) | 235,481  (491) | 244,251  (468) | 279,022  (488) |
| 4 | Excluding individuals who did not have outpatient visits at hospitals for influenza | 406,664  (141) | 74,078  (117) | 71,484  (121) | 87,356  (123) | 87,994  (122) | 85,752  (122) |

**Note:** Our research participants may still have outpatient visits for influenza at non-hospital outpatient facilities. Only hospitals with at least one encounter in the dataset were included by this study for analysis.

**Appendix 5 The procedure of performing the spatial analyses**

**Step 1 Obtaining addresses and coordinates of hospitals**

The addresses of the hospitals were obtained from the online platforms of three information companies that publish a vast repository of Chinese enterprise information. We manually collected the longitudes and latitudes of hospitals from the “Baidu map coordinate extraction system”. Data on the road networks and the administrative boundary maps at a scale of 1:25,000 were extracted from the China National Earth System Science data center. The “Flint” data were derived from the China Remote Sensing Satellite Ground Station of Chinese Academy of Science.

**Step 2 Transforming the geographic coordinates systems**

We transformed the geographic coordinate system of coordinates of hospitals (the BD-09 geographic coordinate system) and the nighttime lights (the D_WGS_1984 geographic coordinate system) into the same geographic coordinate system with the road networks (the China Geodetic Coordinate System 2000). The distribution of the road networks in Changde city was shown in Appendix 3.1.

**Step 3 Creating layers in the ArcMap**

We loaded a satellite image of Changde city that contains boundaries of all its districts/counties into the ArcMap. After that we added all the coordinates of hospitals, the nighttime lights data, and the road networks data into the ArcMap. These geospatial data were then projected onto the map using the projected coordinate system CGCS2000_3_Degree_GK_CM_111E. We used the 3-degree Gauss-Kruger zone 37 since its longitude ranges from 109.5 to 112.5 within which Changde city is located.

**Step 4 Performing the OD cost matrix analysis and Service Area analysis**

We dissolved the roads of the same type that were not separated by the junction points before converting all features to lines. A comparison of the distribution of the road networks before and after data processing was shown in Appendix 3.2. Then we created a network dataset, the required data format to perform the OD cost matrix analysis. The output of the OD cost matrix analysis is a distance matrix of all hospitals. Afterwards, we generated the catchment area for each hospital to show locations which can be reached within certain market radii (in our study, 5km, 10km, and 15km). The catchment area for hospitals with different market radii can be seen in Appendix 3.3.

**Step 5 Performing the Buffer analysis and the Zonal Statistic analysis**

We created a buffer zone for each hospital based on the size of each hospital. Data on the nighttime lights were stored in raster format that is arranged in a regular grid of cells. We thus performed the Zonal Statistics analysis to calculate the annual intensity of nighttime lights for each hospital.

**Appendix 5.1 The distribution of the road networks in Changde city**

**
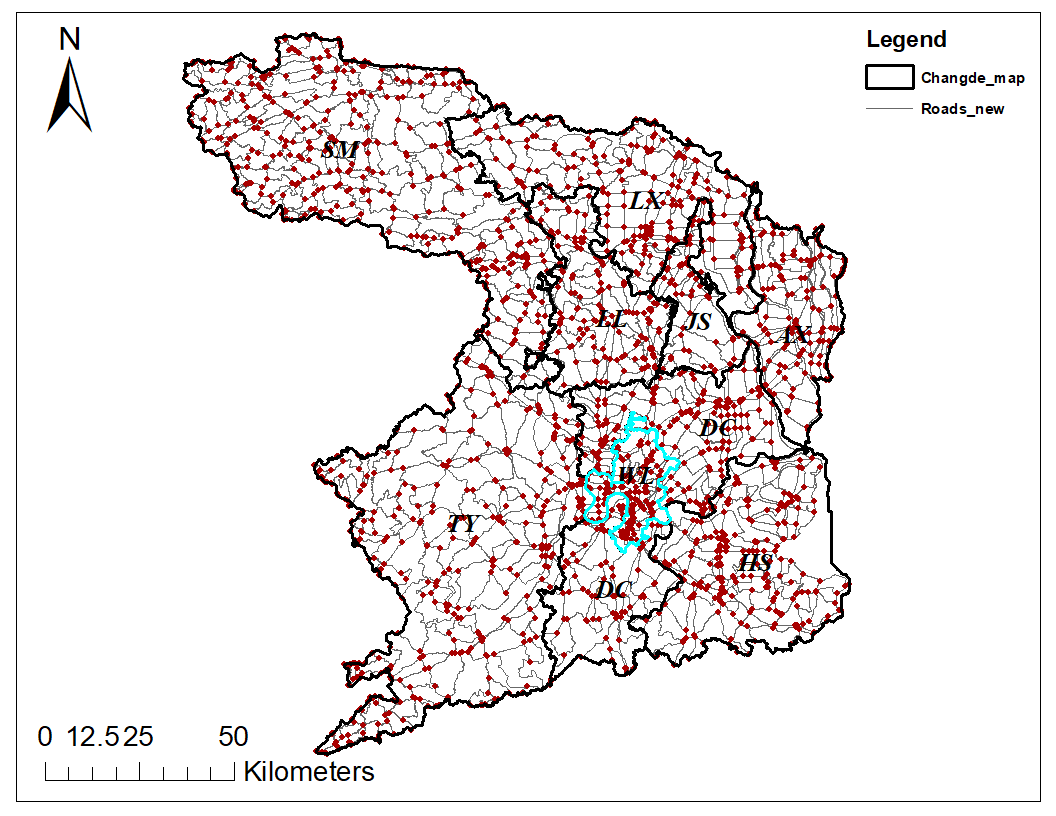
**

**Note:** The figure was made by the author.

**Appendix 5.2 The comparison of the road networks before and after data processing**

(Upper: before data processing; Lower: after data processing)

**
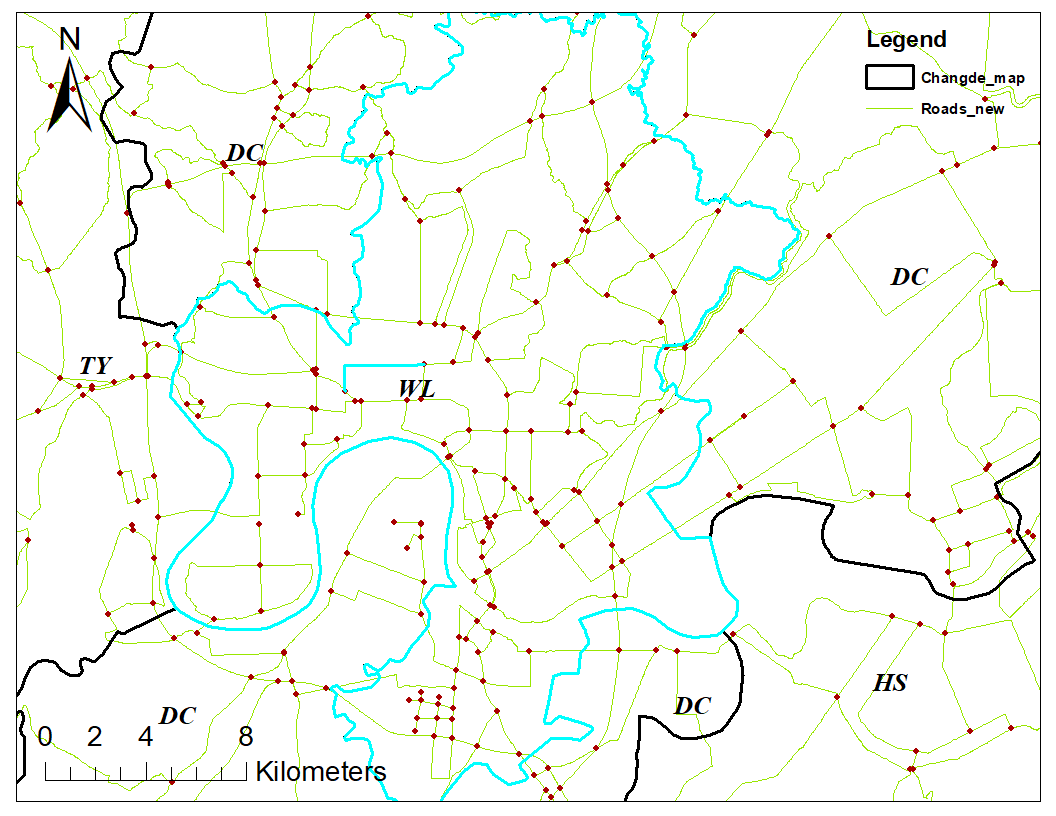

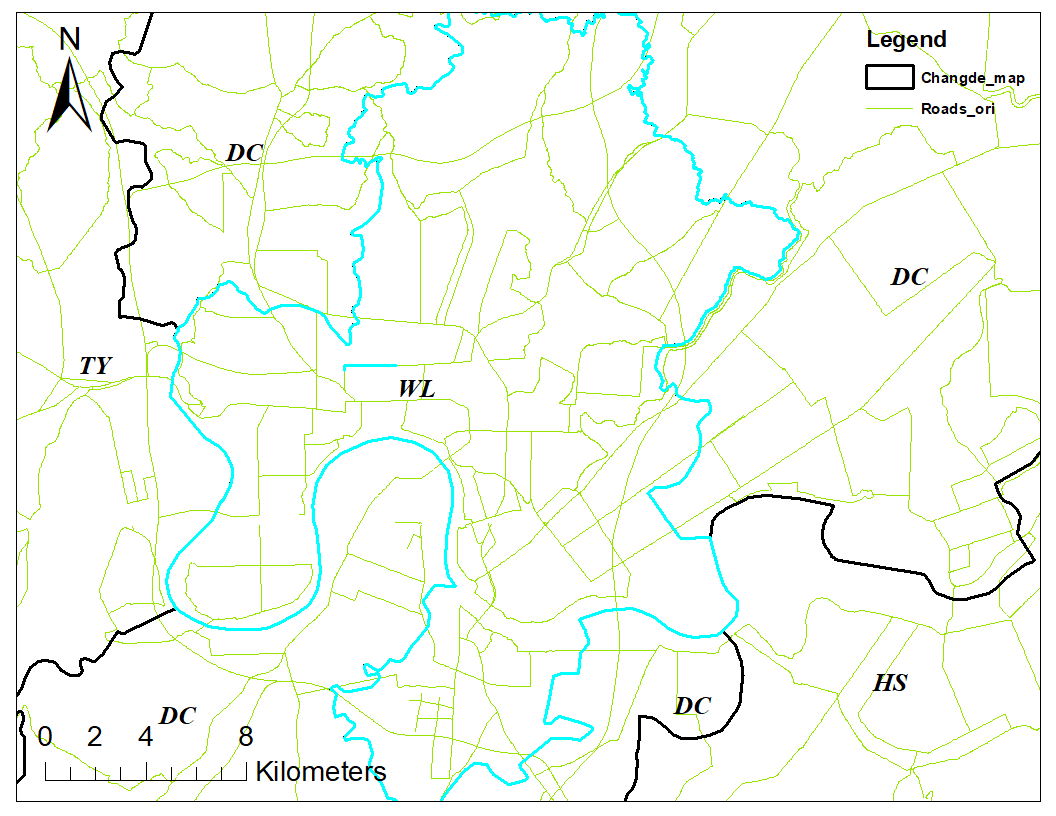
**

**Note:** The figure was made by the author.

**Appendix 5.3 The distribution of hospital markets with different radii**

(Upper: 5 km; Middle: 10 km; Lower: 15 km)

**
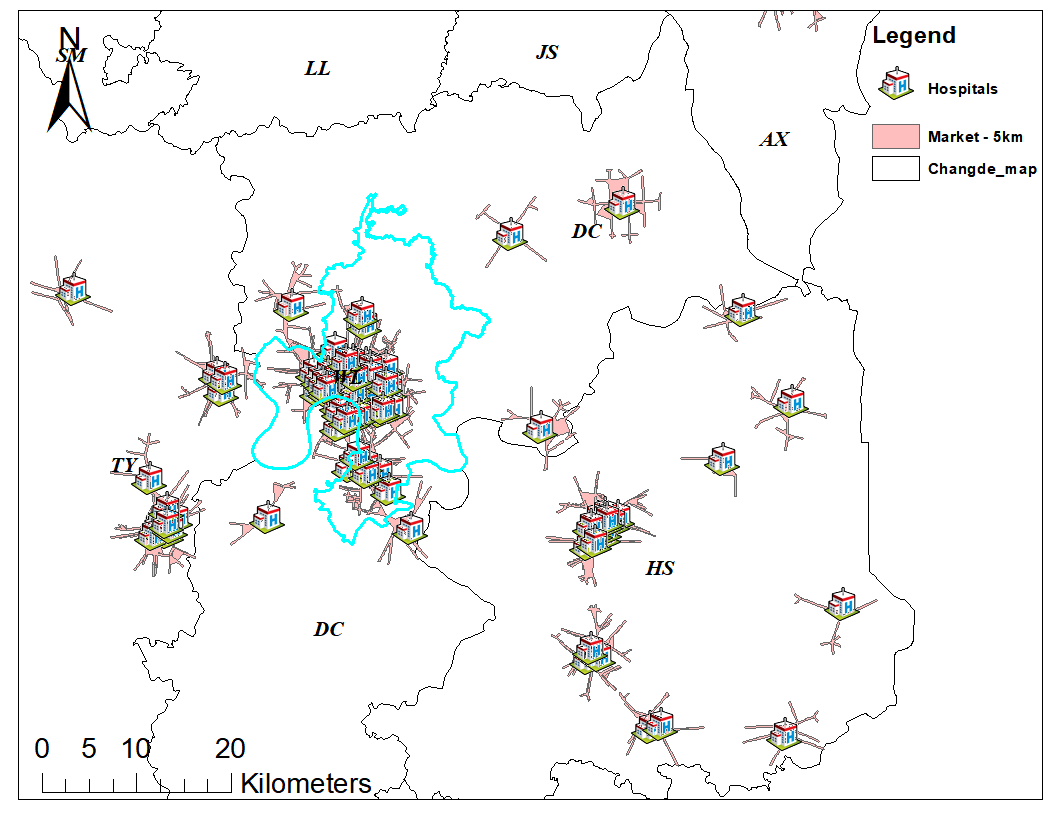
**

**
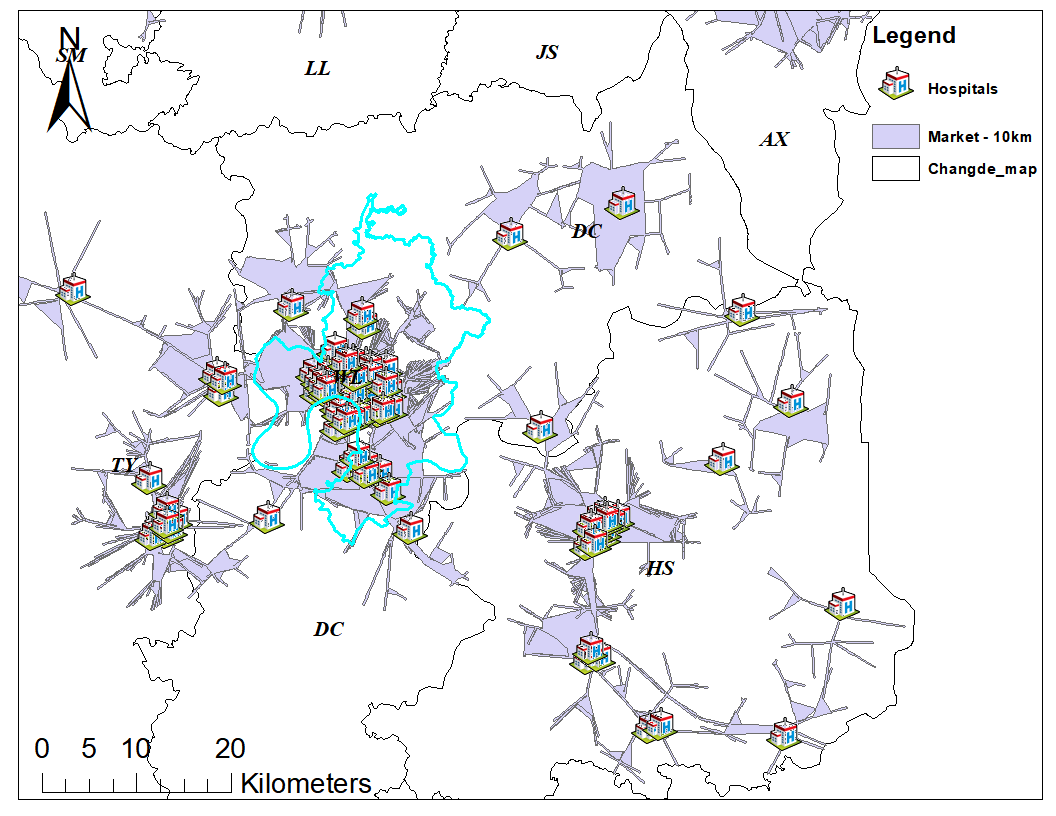
**

**
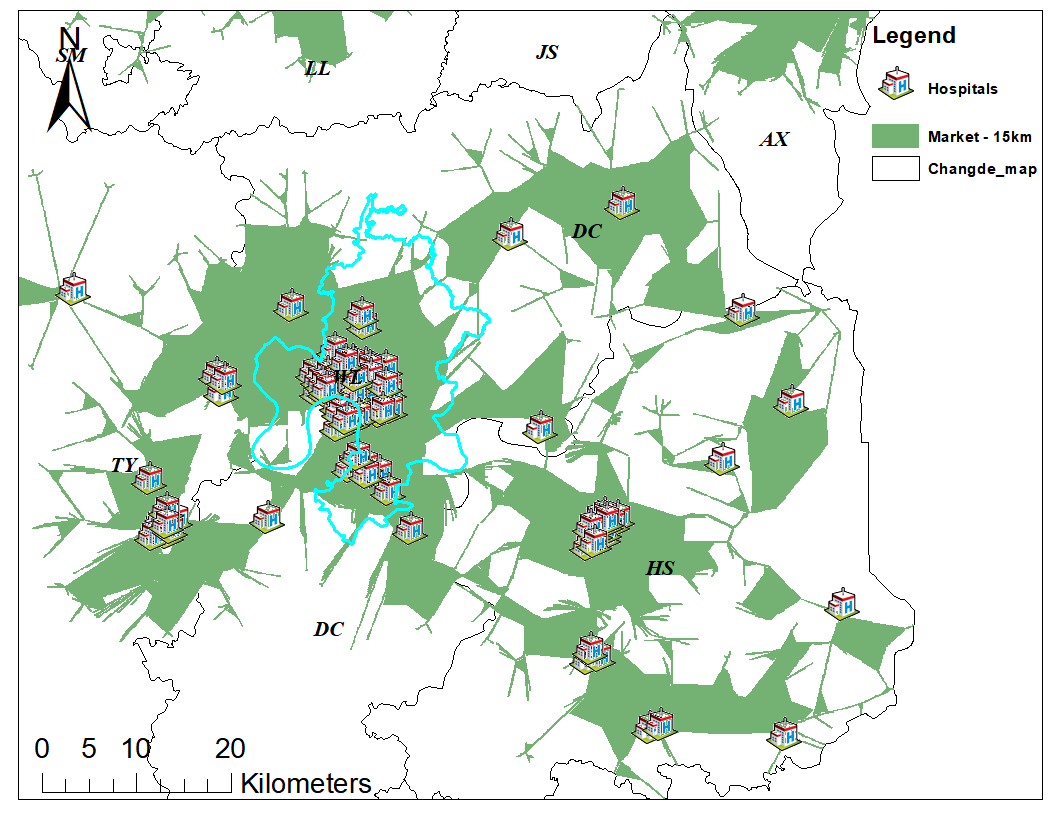
**

**Note:** The figure was made by the author.

**Appendix 6 The annual mean HHI values for the sampled hospitals from 2015 to 2019**

**Note:** The figure was made by the author.

**Appendix 7 The percentage of patients who had follow-up encounters from 2015 to 2019**

**Note:** The figure was made by the author.

**Appendix 8 The impacts of hospital competition on the odds of having follow-up encounters at any healthcare facilities (heterogeneity analysis)**

**Note:** The figure was made by the author.

**Appendix 9 The impacts of hospital competition on the odds of having follow-up encounters at any outpatient facilities (heterogeneity analysis)**

**Note:** The figure was made by the author.

**Appendix 10 The impacts of hospital competition on the odds of having follow-up encounters at any hospital outpatient departments (heterogeneity analysis)**

**Note:** The figure was made by the author.

**Reference**

1. Siciliani, L., M. Chalkley, and H. Gravelle, *Policies towards hospital and GP competition in five European countries.* Health Policy, 2017. **121**(2): p. 103-110.

2. Deng, C. and J. Pan, *Hospital competition and the expenses for treatments of acute and non-acute common diseases: Evidence from China.* BMC Health Services Research, 2019. **19**(1): p. 739.

3. Croes, R.R., Y.J.F.M. Krabbe-Alkemade, and M.C. Mikkers, *Competition and quality indicators in the health care sector: Empirical evidence from the Dutch hospital sector.* The European Journal of Health Economics, 2018. **19**(1): p. 5-19.

4. Hehenkamp, B. and O.M. Kaarbøe, *Location choice and quality competition in mixed hospital markets.* Journal of Economic Behavior & Organization, 2020. **177**: p. 641-660.

5. Giuseppe, M., G. Hugh, and S. Luigi, *Hospital competition and quality for non-emergency patients in the English NHS.* The RAND Journal of Economics, 2021. **52**(2): p. 382-414.

6. Brekke, K.R., L. Siciliani, and O.R. Straume, *Hospital competition and quality with regulated prices.* Scandinavian Journal of Economics, 2011. **113**(2): p. 444-469.

7. Laura, L. and L. Rosella. *Oligopolistic competition for the provision of hospital care*. SSRN Working Paper 2017 [cited 2022; Available from: <https://papers.ssrn.com/sol3/papers.cfm?abstract_id=2893575>.
